# APOEε4 attenuates early TREM2-mediated microglial responses and influences disease trajectories in dementia with Lewy bodies

**DOI:** 10.21203/rs.3.rs-10429506/v1

**Published:** 2026-07-31

**Authors:** Estrella Morenas Rodríguez, Pablo Zaragoza-Ballester, Daniel Alcolea, Diana Esteller-Gauxax, Brigitte Nuscher, Sara Chacón, Álex Fernández-León, Íñigo Rodríguez-Baz, Alexandre Bejanin, Elena Vera, Lidia Vaqué-Alcázar, Maria Franquesa-Mullerat, Isabel Sala-Matevera, Kai Schlepckow, Adolfo Gómez-Grande, Sebastián Ruiz Solís, Alberto Lleó, Valle Camacho, Mircea Balasa, Juan Fortea, Christian Haass

**Affiliations:** Instituto de Investigación Hospital 12 de Octubre; Hospital de la Santa Creu i Sant Pau; Hospital de Sant Pau - IIB Sant Pau; Alzheimer’s disease and other cognitive disorders Unit. Service of Neurology, Hospital Clínic de Barcelona. Fundació Recerca Clínic Barcelona-IDIBAPS. Department of Medic; Ludwig-Maximilians-University Munich; Hospital Universitario 12 de Octubre (Imas12) Research Institute; Hospital de la Santa Creu i Sant Pau; Sant Pau Memory Unit, Department of Neurology, Hospital de la Santa Creu i Sant Pau, Institut de Recerca Sant Pau (IR SANT PAU); Institut de Recerca Sant Pau - Centre CERCA Sant Quintí, 77-79 - 08041 Barcelona; Hospital de la Santa Creu i Sant Pau; University of Barcelona; Sant Pau Memory Unit, IR SANT PAU, Hospital de la Santa Creu i Sant Pau, Barcelona, Spain; Hospital de la Santa Creu i Sant Pau; German Center for Neurodegenerative Diseases (DZNE); Hospital Universitario 12 de Octubre; Hospital Universitario 12 de Octubre; Hospital de la Santa Creu i Sant Pau; Hospital Sant Pau; Alzheimer's disease and other cognitive disorders Unit. Service of Neurology, Hospital Clínic de Barcelona. Fundació Recerca Clínic Barcelona-IDIBAPS. Centro de Investiga; Sant Pau Memory Unit, Hospital de la Santa Creu i Sant Pau - Biomedical Research Institute Sant Pau-Universitat Autònoma de Barcelona; Biomedical Center (BMC), Ludwig-Maximilians-Universität

**Keywords:** DLB, microglia, TREM2, AD, APOE, cognitive decline, neurodegeneration, synucleinopathy

## Abstract

Dementia with Lewy bodies (DLB) is characterized by marked biological heterogeneity, only partly explained by the frequent presence of Alzheimer’s disease (AD) copathology. Whether microglial responses contribute to this heterogeneity and how they are modulated by APOE genotype remain poorly understood. Using a specific immunoassay targeting cleaved soluble TREM2 (sTREM2), we investigated TREM2-dependent microglial responses across two independent DLB cohorts (n = 129) with molecular biomarker profiling and longitudinal follow-up. sTREM2 showed stage-dependent associations with AD-related biomarkers, being associated with amyloid-related changes during prodromal DLB and predominantly to tau-related markers at dementia stage. *APOEε4* carriers exhibited approximately two-fold lower sTREM2 levels than non-carriers specifically during prodromal DLB, independently of AD copathology, with more pronounced effects observed in women. Longitudinal analyses showed that higher baseline sTREM2 levels during prodromal DLB were associated with slower cognitive decline independently of AD-related biomarkers. These findings suggest that *APOEε4* attenuates early TREM2-mediated microglial responses in DLB through mechanisms beyond AD copathology. Together, our results identify the APOE–TREM2 axis as a potential contributor to disease heterogeneity and support stage-specific, biomarker-guided therapeutic strategies targeting TREM2 signaling in Lewy body disorders.

## Introduction

Dementia with Lewy bodies (DLB) is a common neurodegenerative dementia that represents a major challenge from both research and clinical perspectives due to its marked biological and phenotypic heterogeneity as well as its overlap with Alzheimer’s disease (AD) and Parkinson disease (PD). This complexity limits our understanding of the biological processes underlying the highly variable disease trajectories that characterize DLB and the interrelated factors that modulate them. Neuropathologically, DLB is defined by the aggregation of α-synuclein into intracellular inclusions forming Lewy bodies ^1^, yet this process frequently coexists with AD-related amyloid-β and tau pathology. This overlap suggests a complex interplay between α-synuclein and AD-related processes, underlying the high prevalence and variability of AD copathology in Lewy body diseases which ranges from absent to levels comparable to primary AD ^2,3^. This wide range of copathology partially contributes to the observed heterogeneity in DLB, as it has been associated with faster progression, worse cognitive outcomes, and increased neuropsychiatric burden ^4-6^. However, we do not completely understand the factors modulating the wide range of AD copathology in DLB and, moreover, which biological contributors, beyond AD copathology, underly the diversity of DLB clinical trajectories.

AD neuropathology is strongly related to the ε4 allele of the *apolipoprotein E* gene (*APOE*), the strongest genetic risk factor for sporadic AD ^7^. It has been shown that *APOEε4* carriage contributes to AD pathogenesis by modulating multiple pathways beyond amyloid-β metabolism, such as lipid transport ^8,9^, synaptic plasticity ^10^, or neuroinflammation ^11-14^. *APOEε4* has also been described as a genetic risk factor for DLB ^15^. Its association with a higher frequency of AD copathology in DLB is consistently reported ^16,17^, also contributing to greater Aβ and tau burden ^18^. Regarding α-synuclein pathology, *APOEε4* carriage has also been related to a higher pathological burden, with some studies reporting a mediation by AD copathology in this relationship ^17,19^, while others show a direct relationship between *APOEε4* carriage and α-synuclein burden independent of AD copathology ^20,21^. At a clinical level, *APOEε4* has been also associated with more severe cognitive symptoms in DLB, generally attributed to the higher frequency of AD copathology in carriers ^17,19-22^. However, whether *APOEε4* effects are primarily mediated through AD copathology or through its influence on other biological pathways remains unclear.

*APOE* genotype has been implicated in the modulation of microglial phenotypes across aging and AD-related neuropathological changes ^23^. At the molecular level, APOE acts as an endogenous ligand of Triggering Receptor Expressed on Myeloid Cells 2 (TREM2), a microglia-enriched receptor that regulates phagocytosis, lipid sensing, and immune responses ^24,25^. Notably, some studies suggest that APOE isoforms differ in their binding affinity to TREM2, pointing to isoform-specific effects on microglial response ^25,26^. TREM2 activation is essential for immune function in the brain, promoting proliferation, tropism and survival of microglia ^27^. In the context of amyloid-β pathology, TREM2 response has been shown to exert protective effects by facilitating microglial clustering around amyloid-β plaques, thereby limiting fibrillar expansion and associated neurotoxicity ^28^. Experimental studies in mouse models further suggest that the APOEε4 isoform differentially modulates this pathway, impairing microglial responses to amyloid-β pathology and reducing plaque-associated microglial coverage ^29^.

Soluble TREM2 (sTREM2), a cleavage product of TREM2, has emerged as a fluid biomarker reflecting TREM2-dependent microglial response across neurodegenerative disorders ^30-32^. In the context of AD, longitudinal biomarker-based studies indicate that early amyloid-β aggregation acts as a primary trigger of the TREM2-mediated microglial response ^33,34^. Furthermore, increased early TREM2-dependent microglial response, as measured by sTREM2 in cerebrospinal fluid (CSF), has been associated with an attenuation of amyloid-β deposition, neurodegeneration and cognitive decline (Morenas-Rodríguez et al., 2022; Ewers et al., 2019). Beyond AD, longitudinal sTREM2 increase in CSF has also been associated with early synaptic dysfunction throughout aging in the absence of underlying amyloid-β pathology, supporting a broader role of TREM2-dependent responses in neurodegeneration independent of AD-related neuropathological changes ^35^. Notably, synaptic dysfunction represents an early and central event in the pathological cascade of Lewy body diseases, and elevated sTREM2 levels have been already reported in Parkinson’s disease and DLB ^36,37^, suggesting a biological role of TREM2-dependent microglial response also in the context of synucleinopathies.

Together, these observations highlight sTREM2 as a dynamic marker of microglial responses at the intersection of amyloid-β and α-synuclein-related pathological processes, further modulated by *APOE* genotype. Here, we investigate the interplay between *APOE* genotype and TREM2-dependent microglial response in DLB, and its relationship with AD-related pathological changes. Studying two well-characterized clinical cohorts of DLB patients integrating molecular biomarker profiling and clinical longitudinal data, we assess how *APOEε4* carriage status influences microglial responses, as captured by CSF sTREM2, and how these interactions relate to AD copathology and disease progression across DLB stages. This approach aims to determine whether TREM2-dependent microglial responses and its modulation by *APOE* genotype influence DLB trajectories and to identify the factors that modulate these responses, providing a biologically grounded framework to better understand disease variability and inform biomarker-driven stratification and potential therapeutical targets in Lewy body disorders.

## Methods

### Participants

As a discovery cohort, we studied a subset of participants from the Sant Pau Initiative Neurodegeneration (SPIN) cohort ^38^. The SPIN cohort is a multimodal research cohort for biomarker discovery and validation that includes participants recruited from 2013 onwards, with different neurodegenerative dementias, mild cognitive impairment and cognitively normal controls. For the present study, we included patients with a clinical diagnosis of prodromal DLB (prodDLB, n = 39) and mild dementia DLB (demDLB, n = 37). We also included 40 cognitively healthy volunteers selected from the SPIN cohort. All participants received a clinical and formal neuropsychological assessment and underwent lumbar puncture to obtain CSF. DLB patients were evaluated using a previously reported clinical protocol ^32,39^, which includes a structured questionnaire about DLB signs and symptoms. Patients with demDLB met consensus criteria for probable DLB ^40^. Patients with prodDLB met research criteria for prodromal DLB ^41^. A subset of 67 patients had available longitudinal data at the time of the analyses.

To confirm the results, we repeated the analyses in an independent replication cohort, the CLIMB-DEM cohort. The CLIMB-DEM cohort is a single-centre observational cohort recruited at a tertiary memory clinic and formed by subjects referred for cognitive impairment between 2009 and 2025, with available CSF samples at the time of their initial evaluation and at least 6 months of follow-up. CLIMB-DEM cohort recruited 650 participants, who underwent a neurological assessment, neuropsychologist testing, and structural neuroimaging. All subjects were followed up with clinical visits generally every 6 months and clinical groups were based on the clinical diagnoses at last-follow-up visit and based on current consensus criteria. Within the CLIMB-DEM cohort, 92 participants were finally diagnosed with DLB, 53 of whom met criteria for prodromal DLB and were available for cross-sectional analyses. Longitudinal data were not available for this cohort.

### CSF biomarker quantification.

For the discovery cohort (SPIN cohort) CSF was obtained by lumbar puncture as previously described ^42,43^. CSF was collected and processed in polypropylene tubes following international recommendations. The first 2 ml of CSF were transferred to the general laboratory for cell count, and analysis of glucose and protein levels. A further 10 ml were transferred to the memory unit research laboratory where samples were processed (centrifuged 2000 g at 4 C, during 10 min) and aliquoted within the first two hours after the lumbar puncture. Aliquots were stored at − 80°C until analysis. For the CLIMB-DEM cohort, CSF was obtained at the first evaluation and collected and processed in polypropylene tubes according to international recommendations. The first 4 mL of CSF were transferred to the clinical laboratory for cell count, glucose and protein measurements, quantification of Alzheimer's disease core biomarkers, and performance of the alpha-synuclein seed amplification assay. An additional 5 mL were transferred to the Alzheimer’s unit and other cognitive disorders group laboratory, where the samples were processed (centrifuged at 2,000 × g for 10 min at 4°C) and aliquoted within 2 h of lumbar puncture. The aliquots were stored at − 80°C until analysis.

#### AD biomarkers.

For quantification of AD core biomarkers (β1–42, p-tau181, and t-tau), we used available ELISA kits following manufacturer’s recommendations ^44^. Internal cut-offs were validated in a sample of patients who underwent amyloid PET ^44^. Amyloid positive status was defined as a CSF Aβ1–42 concentration below 550 pg/mL, whereas tau positive status was defined as a CSF p-tau181 concentration above 61 pg/mL. For the CLIMB-DEM cohort, AD core biomarkers were measured with Lumipulse following the manufacturer’s instructions (Fujirebio, Belgium). Amyloid positive status was defined as CSF amyloid β1–42 below 600 pg/mL, meanwhile tau status was defined as CSF p-tau181 above 65 pg/mL ^45^.

##### Cleaved soluble TREM2 (sTREM2)

For quantitative determination of sTREM2 concentrations in CSF, we used a Meso Scale Discovery-based immunoassay which has been described previously ^31,33,46,47^. In brief, to avoid detection of soluble TREM2 variants generated by alternative splicing, this immunoassay uses a detection antibody (1H3) that is directed against the neo-epitope on the C-terminus of soluble TREM2, derived by ADAM10 and ADAM17 mediated cleavage of the full-length TREM2 protein. The use of this specific antibody allowed us to selectively measure soluble TREM2 derived from the signalling competent cell-surface precursor. Samples coming from SPIN Cohort were measured in the Haass Lab, and samples from CLIMB-DEM were measured in Hospital 12 de Octubre following the same procedures.

All samples were measured in duplicate and randomized across plates, including the same internal standard samples (IS) across plates to ensure inter-assay and inter-plate reproducibility. Measurements with a coefficient of variation (CV) > 15% were repeated or excluded from subsequent analyses. Samples from the SPIN cohort were measured in two different days. Mean intraplate CV was 4.33% and interplate CV was 12.69%. To minimize batch effects in this cohort, a correction factor was applied as previously described ^47^. Samples from the CLIMB-DEM cohort were measured on the same day. For CLIMB-DEM cohort, intraplate CV was 3.50%; and interplate CV was 8.10%. Interplate CV was calculated from two different IS for the SPIN cohort and three different IS from the CLIMB-DEM cohort, each generated from pooled CSF samples obtained from independent clinical leftovers.

###### *APOE* genotyping.

In the SPIN cohort, DNA was extracted from whole blood samples using the DNeasy^®^ *Blood &Tissue* kit (Qiagen) and *APOE* genotype was determined by direct DNA sequencing of exon 4 and visual analysis of the resulting electropherogram was performed to identify the two coding polymorphisms that encode the three possible APOE isoforms ^38^. In CLIMB-DEM, APOE genotype was determined through analysis of 2 single-nucleotide variations (formerly single-nucleotide polymorphisms) (rs429358 and rs7412) by Sanger sequencing ^45^.

###### Amyloid PET procedure.

Amyloid PET was available in 37 patients from the SPIN cohort ^38^. In those cases, amyloid PET was performed according to the Sant Pau Hospital protocol. Briefly, PET/CT imaging was performed on a PHILIPS GEMINI TF PET/CT scanner 60 minutes after intravenous administration of 370 MBq (10 mCi) of [18F]Florbetapir. The imaging protocol comprised a low-dose CT acquisition (100 mA, 130 kVp) for attenuation correction and anatomical localization, followed by a three-dimensional time-of-flight (3D TOF) PET acquisition. PET images were reconstructed using the LOR-RAMLA iterative reconstruction algorithm with a 256 mm field of view, a 128 × 128 image matrix, 89 transaxial slices, and an isotropic voxel size of 2 × 2 × 2 mm. In addition, T1-weighted magnetic resonance imaging (MRI) images were acquired at Hospital del Mar (Barcelona, Spain) within one year of the PET scans. The acquisition parameters were: echo time (ms) = 3.8, repetition time (ms) = 3.2, Flip angle (°) = 8, number of slices = 160, slice thickness (mm) = 1, field of view (mm^2^) = 256x256, voxel resolution (mm) = 0.94x0.94x1. PET images were first co-registered to the corresponding T1-weighted image in native space using a rigid-body transformation (6 degrees of freedom) computed with FreeSurfer. The T1-weighted images were then registered to the MNI152 standard space using ANTs (Advanced Normalization Tools). The registration pipeline included three sequential steps: a Rigid registration, an Affine transformation, and a non-linear SyN registration, using a multi-resolution approach to improve accuracy. The resulting transformation was then applied to warp both the PET image and its associated brain mask (defined as voxels with PET values > 0) into MNI space, allowing all images to be compared in a common reference frame. Standardized Uptake Value Ratios (SUVRs) were computed in MNI space using tracer-specific reference and target regions of interest (ROIs). The Centiloid project standard ROIs were applied, using the whole cerebellum as reference region, and a cortical mask as target. All ROIs were resliced to match the 2mm MNI template resolution using nearest-neighbour interpolation prior to SUVR computation.

## Statistical analysis.

Normality of the variables was tested by Shapiro-Wilk test. Non-normally distributed variables (sTREM2, Aβ42, p-tau, t-tau) were log-transformed to achieve a normal distribution and all the analyses were performed with the log-transformed values. For some analysis, we stratified patients according to TREM2-dependent microglial response using sTREM2 median. Patients with high TREM2-dependent microglial response were defined as those with a sTREM2 levels higher than the median of sTREM2, while patients with low TREM2-dependent microglial response were defined as those with a sTREM2 levels lower than the median of sTREM2. For the stratification according to the A/T scheme, we use CSF Aβ42 levels for amyloid-β status and CSF p-tau levels for tau status. Cohort-specific cutoff values were applied in each cohort. In addition, we also used amyloid PET for amyloid-β status, using the whole cerebellum as reference region and a cut-off value of 1.17 was applied ^48^. Sex was defined based on participants' self-report.

Cross-sectional analyses focused on descriptive characteristics at baseline, including demographic variables and biomarker values, were performed by X^2^ tests for categorical variables, and ANOVA or ANCOVA tests for continuous variables. ANCOVA was adjusted for the effect of sex and was done to study the differences between biomarkers at baseline across the diagnostic groups in the SPIN cohort. Partial Pearson Product-Moment correlations controlled by sex were used to test the association between continuous variables. To better account for the independent relationship between different baseline biomarkers and CSF sTREM2, we performed linear regression models (LRM). We used CSF sTREM2 levels as the main dependent variable and performed two types of models: firstly, adjusted for sex and including an amyloid-aggregation variable (CSF Aβ42 or uptake in the amyloid PET) and CSF p-tau181 as covariates, and secondly, further adjusted for *APOEε4* carriage status. Furthermore, we repeated the models including interaction terms (Aβ42*APOEε4, p-tau*APOEε4 and sex*APOEε4). These analyses were performed stratifying patients according to clinical stages of DLB. As age in the discovery cohort and sex in the replication cohort did not materially influence the models, these covariates were excluded from the final models in their respective cohorts.

To study the influence of TREM2-dependent microglial response and *APOE* genotype on cognitive evolution, we included those patients with more than one-year of follow-up in the longitudinal analysis. First, we evaluate the association between sTREM2 and MMSE cross-sectionally at baseline by linear regression models, adjusted for age, CSF Aβ42 and p-tau181 at baseline. *APOEε4* carriage status was subsequently added as an additional independent variable in the model to assess its independent association with baseline cognition. Secondly, we evaluated the association between sTREM2 and *APOE* genotype and time to progression from baseline evaluation to mild dementia (GDS 4) in the case of prodDLB patients and to severe dementia (GDS 6) in the overall cohort by linear regression models, adjusted for age, CSF Aβ42 and p-tau181 levels at baseline. *APOEε4* carriership was additionally included afterwards. And finally, we studied MMSE score variation over time by longitudinal linear mixed effects (LME) models, adjusted for age, and CSF Aβ42 and p-tau181 at baseline. This LME models were used to assess the effect of baseline biomarkers (predictor) on the longitudinal change of the MMSE score. The level of significance was set at 5% (α = 0.05). All statistical analyses were performed using R software version 4.3.3 (2024-02-29 ucrt) for Windows.

### Sensitivity analyses.

To assess the robustness of the association between *APOEε4* carriage and CSF sTREM2 levels, we performed analyses including and excluding CSF sTREM2 extreme values. We defined extreme sTREM2 values per each *APOEε4* carrier status group as CSF sTREM2 measurements below Q1 – 1.5 × interquartile range (IQR) or above Q3 + 1.5 × IQR. We used the IQR method for the detection of outliers considering the non-normal distribution of CSF sTREM2 values. This methodology was applied to the primary analyses in the replication cohort (i.e., the association between *APOEε4* carriage and CSF sTREM2 levels) as it is shown in the main text. The complete set of results, including analyses with and without outlier exclusion and full description of outliers, is presented in the Supplementary Material.

## Results

### Discovery cohort (SPIN).

The SPIN cohort included 76 DLB patients in stage of mild cognitive impairment (prodDLB, n = 39), or mild dementia (demDLB, n = 37), and 40 cognitively healthy volunteers (HV). Table 1 summarizes the demographics and baseline clinical and biomarker data from these participants. Groups did not differ in sex proportion or frequency of *APOEε4* carriage. Age correlated with all CSF AD core biomarkers, while sex only was related to higher levels of CSF p-tau in men compared to women in the HV group (50.33 ± 9.06 vs. 41.87 ± 9.70 pg/mL, p-value = 0.009). *APOEε4* carriers showed lower levels of CSF Aβ42 compared to non-carriers in both prodDLB and demDLB groups (571.22 ± 155.03 vs. 813.68 ± 302.20 pg/mL, p-value = 0.030; and 444.73 ± 123.26 vs. 661.96 ± 201.10 pg/mL, p-value = 0.001; respectively), but no differences were found in tau-related markers depending on the *APOEε4* status. Concerning FBP-PET, we only found differences in prodDLB, where *APOEε4* carriers showed a trend to higher SUVR values compared to non-carriers (1.39 ± 0.18 vs 1.12 ± 0.18, p-value 0.064).

#### DLB patients had higher levels of CSF sTREM2 than HV.

First, we studied whether CSF sTREM2 levels differed across DLB stages and compared with HV and whether they were influenced by sex or age. Both the prodDLB and demDLB groups showed higher CSF sTREM2 levels than the HV group (prodDLB vs HV: 6.11 ± 2.51 vs. 3.87 ± 1.88 ng/mL, p-value < 0.001; demDLB vs HV: 5.29 ± 2.91 vs. 3.87 ± 1.88 ng/mL, p-value = 0.019; Table 1). However, no statistically significant differences were observed between the two DLB groups. CSF sTREM2 levels were not influenced by sex in our study cohort, nor age in the DLB groups. Only the HV group showed a positive partial correlation between CSF sTREM2 levels and age (r = 0.49, p-value = 0.001).

#### The relationship between CSF sTREM2 levels and core AD biomarkers differs depending on the clinical stage of DLB.

Next, we investigated the relationship between CSF sTREM2 levels and AD core biomarkers in DLB groups. We studied the relationship between CSF sTREM2 levels and AD-related markers by linear regression models adjusted for sex to better account for the interrelationship between different AD-related biological processes and microglial activation in the context of DLB. Linear regression models are represented in Supplementary Table 1. In the prodDLB group, CSF sTREM2 levels were independently associated with Aβ42 levels (β = 0.777, p-value < 0.001) and p-tau levels (β = 0.617, p-value = 0.001). In the demDLB group, CSF sTREM2 levels were independently associated with p-tau levels (β = 0.608, p-value < 0.001), but not with Aβ42 levels, in contrast to the prodromal phase of the disease.

**Figure 1:**
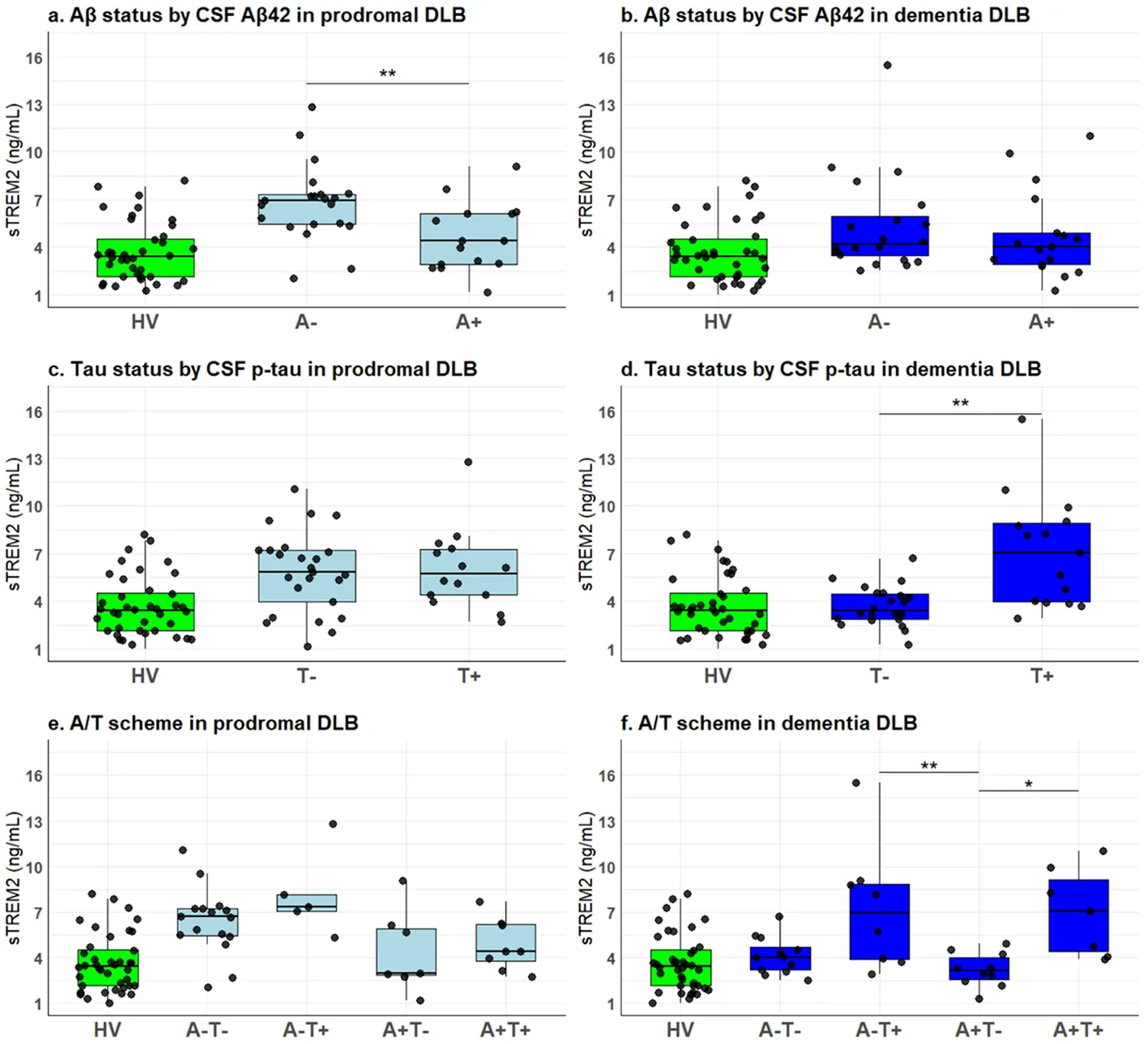
sTREM2 shows disease phase-dependent associations with AD co-pathology biomarkers. Box and whisker plots of the median concentration of cleaved sTREM2 levels according to the A/T scheme in both prodDLB and demDLB groups. **a and b.** Aβ status was defined by ELISA test and based on CSF levels of Aβ42, considering A+ when they were lower than 550 pg/mL. **c and d.** Tau status was defined by ELISA test and based on CSF levels of p-tau, considering T+ when they were higher than 61 pg/mL. **e and f.** Complete A/T scheme considering previous cut-offs. prodDLB=prodromal Dementia with Lewy Bodies. demDLB=Dementia with Lewy Bodies. HV= cognitively healthy volunteers. sTREM2=cleaved soluble TREM2. *p-value < .05 **p-value < .01

Consistent with these findings, stratification according to the A/T scheme revealed stage-specific associations. In the prodDLB group, CSF sTREM2 levels differed according to amyloid status, with higher levels in amyloid negative than in amyloid positive group (6.98 ± 2.41 vs. 4.83 ± 2.16 ng/mL, p-value = 0.009), whereas no differences were observed according to Tau status (Fig. 1). Conversely, in the demDLB group, CSF sTREM2 levels where higher in Tau positive than in Tau negative group (7.31 ± 3.48 vs. 3.91 ± 1.24 ng/mL, p-value < 0.001), while no differences were found according to amyloid status. These findings support a differential association of sTREM2 with AD-related pathology across disease stages in DLB.

In addition, in prodDLB patients, we found an independent association between CSF sTREM2 levels and amyloid uptake measured by SUVR quantification (β = −2.181, p-value = 0.041) (Supplementary Table 2). Nevertheless, when stratifying prodDLB patients according to amyloid status using SUVr quantification, no differences on CSF sTREM2 levels were found between amyloid positive and amyloid negative groups (Supplementary Fig. 1). These finding suggest that the relationship between sTREM2 and amyloid dysmetabolism during prodromal DLB is stronger for soluble amyloid aggregation markers (Aβ42) than for amyloid deposition markers (uptake in the amyloid PET).

#### APOEε4 carriage independently modulate CSF sTREM2 levels in prodromal DLB.

Interestingly, *APOEε4* carriage significantly influenced regression models in prodDLB, but not demDLB (Supplementary tables 2 and 3). In prodDLB patients, *APOEε4* carriage was independent associated with CSF sTREM2 levels (β = −0.381, p-value = 0.029) and improved prediction model in an 8–9% (from 0.435 to 0.516 (r^2^); AIC prodDLB: from 37.16 to 33.59). We also found a trend for an association between CSF levels of sTREM2 and the interaction between *APOEε4* carriage and Aβ42 levels (β[carrier] = 0.874, p-value = 0.097) in prodDLB group, pointing to a stronger relationship between CSF sTREM2 and Aβ42 in prodDLB *APOEε4* carriers. In contrast, *APOEε4* carriage did not influence models predicting CSF sTREM2 levels in demDLB.

**Figure 2:**
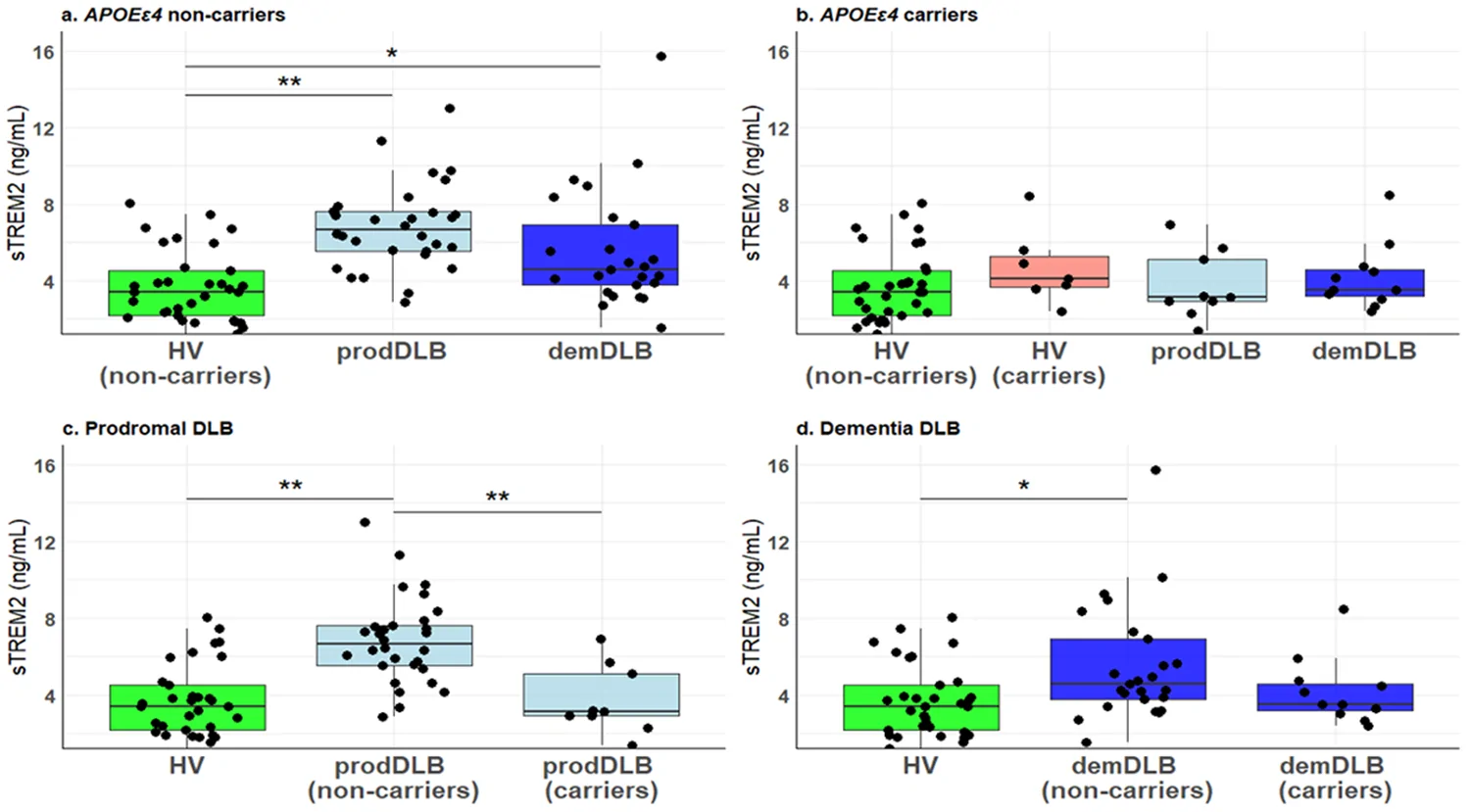
sTREM2 levels according to *APOEε4* carriership status across DLB clinical stages. **a.** Box and whisker plots representing sTREM2 levels in *APOEε4* non-carriers including 33 HV, 30 prodDLB and 25 demDLB patients. **b.** Box-and-whisker plot representing sTREM2 levels in *APOEε4* carriers including 7 HV, 9 prodDLB and 11 demDLB patients, and 33 HV *APOEε4* non-carriers. **c.** Box-and-whisker representing sTREM2 levels in prodDLB group compared to 33 HV. **d.** Box-and-whisker plot of sTREM2 levels in demDLB group, compared to 33 HV. *p-value < 0.01 **p-value < 0.001. DLB = Dementia with Lewy bodies. prodDLB = prodromal DLB, demDLB= dementia DLB. HV= cognitively healthy volunteers. sTREM2 = cleaved soluble TREM2.

**Figure 3:**
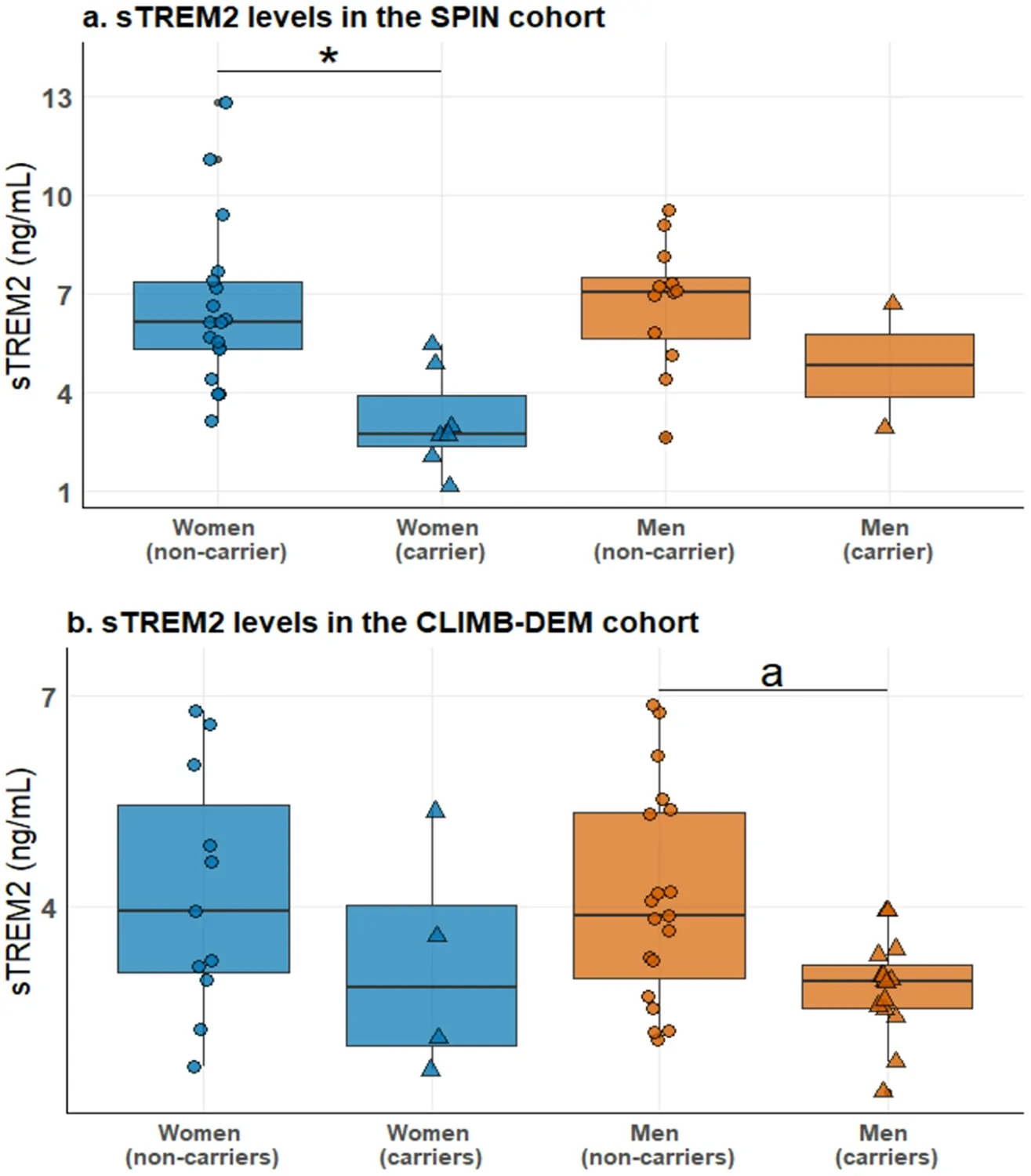
Differences in sTREM2 levels according to sex and *APOEε4* carriership status. **a.** Box-and-whisker plot of sTREM2 levels according to *APOEε4* status stratified by sex (women -shown in blue- and men -shown in orange-) in the prodDLB group of the SPIN cohort. **b.** Box and whisker plots of sTREM2 levels according to *APOEε4* status stratified by sex (women -shown in blue- and men -shown in orange-) in the CLIMB-DEM cohort. Triangles represent *APOEε4* carriers. Circles represent *APOEε4* non-carriers. sTREM2 = cleaved soluble TREM2. *p-value < 0.001. ^a^p-value=0.054

Based on these findings, we examined CSF sTREM2 levels across diagnostic groups stratified by *APOEε4* carriership to assess the influence of *APOE* on the TREM2-dependent microglial response across DLB stages. Interestingly, in prodDLB, *APOEε4* non-carriers exhibited twice the levels of sTREM2 compared to *APOEε4* carriers (6.83 ± 2.25 vs 3.72 ± 1.79 ng/mL, p-value < 0.001) (Fig. 2). This difference was more pronounced in women (women: 6.77 ± 2.49 vs 3.35 ± 1.53 ng/mL, p-value < 0.001; men: 6.91 ± 1.95 vs 5.03 ± 2.68 ng/mL, p-value = 0.582, Fig. 3). Conversely, CSF sTREM2 levels did not significantly differ depending on *APOEε4* carriage in demDLB (5.54 ± 3.06 vs 4.18 ± 1.75 ng/mL, p-value = 0.179), and no differences were observed neither according to sex. Notably, CSF sTREM2 levels in *APOEε4* carriers from the DLB-groups did not differ from CSF sTREM2 levels in HV (Fig. 2), suggesting a lack of sTREM2 elevation in this subgroup of DLB patients.

#### APOEε4 carriage is independently related to a faster clinical decline in DLB.

We, then, examined the impact of *APOEε4* carriage on clinical progression in DLB. *APOEε4* carriers showed a trend toward lower baseline MMSE scores at baseline in the whole cohort (β[carrier]= −2.291, p-value = 0.059, Supplementary table 4). *APOEε4* carriage was also associated with a faster progression to mild dementia (β[carrier]= −3.424, p-value = 0.013) and a trend for a faster progression to severe dementia (β[carrier]= −2.926, p-value = 0.07, Supplementary table 5 and 6) among prodromal DLB patients. In addition, *APOEε4* carriage was independently associated with a faster longitudinal MMSE decline after adjustment for AD-related biomarkers, (β[time*APOEε4 carrier] = −0.949, p-value = 0.011) in the whole DLB group.

#### Higher baseline CSF levels of sTREM2 during the prodromal phase of DLB are related to subsequent slower cognitive decline over time.

**Figure 4:**
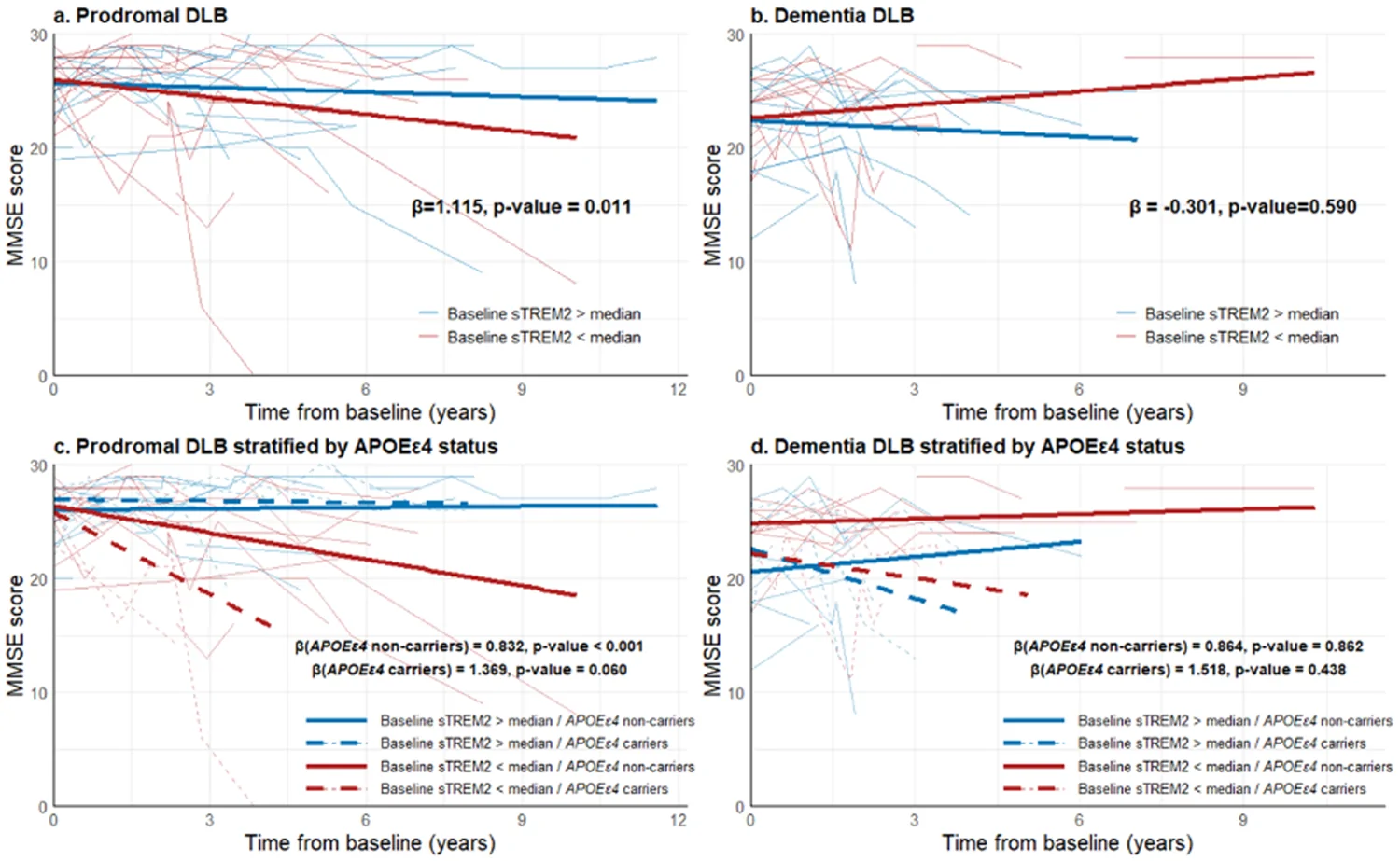
Association between sTREM2 levels at baseline and subsequent cognitive evolution. We show longitudinal cognitive evolution from baseline, as measured by MMSE scores, in prodromal and dementia DLB patients stratified according their sTREM2 levels at baseline for illustrative purposes only (above the median -shown in green-, and below the median -shown in red-). **a.** In prodromal DLB, higher sTREM2 levels at baseline were related to a subsequent slower cognitive decline (β[time*baseline sTREM2] = 1.115, p-value = 0.011) **b.** We did not find any relationship between sTREM2 levels at baseline and subsequent cognitive evolution in dementia DLB patients (β[time*baseline sTREM2] = −0.301, p-value = 0.590). **c.** Cognitive evolution in prodromal DLB stratified by *APOEε4* status showed a slower MMSE decline over time in *APOEε4*non-carriers (β[time*baseline sTREM2] = 0.832, p-value < 0.001). In *APOEε4*carriers, higher baseline sTREM2 levels showed a trend toward a slower MMSE decline (β[time*baseline sTREM2] = 1.369, p-value = 0.060). **d.** Cognitive decline in mild dementia DLB stratified by *APOEε4* status did not showed any relationship with sTREM2 levels at baseline (*APOEε4* carriers: β[time*baseline sTREM2] = 1.518, p-value = 0.438; *APOEε4* non-carriers: β[time*baseline sTREM2] = 0.864, p-value = 0.862). DLB= Dementia with Lewy Bodies. sTREM2 = cleaved soluble TREM2.

To assess the impact of TREM2-dependent microglial response on the clinical evolution of DLB, we next studied whether CSF sTREM2 levels at baseline were related to different disease trajectories. To this aim, we performed lineal mixed models, adjusted for age, Aβ42 and p-tau, and stratified by clinical stage. Notably, in prodDLB patients, higher CSF sTREM2 levels at baseline were associated to a slower decrease in MMSE score (β[time*baseline sTREM2] = 1.115, p-value = 0.011). Next, we stratified prodDLB patients according to APOEε4 status. In *APOEε4* prodDLB non-carriers higher CSF sTREM2 also were associated with a slower MMSE decline over time (β[time*baseline sTREM2] = 0.832, p-value < 0.001), while in *APOEε4* prodDLB carriers, higher baseline CSF sTREM2 levels showed a trend for an association with a slower MMSE decline (β[time*baseline sTREM2] = 1.369, p-value = 0.060), despite the low n-number available for longitudinal analysis in this subset of patients (Fig. 4). This association was not found in demDLB (β[time*baseline sTREM2] = −0.301, p-value = 0.590). These results suggest that a stronger TREM2-dependent response during early symptomatic phases of DLB might be beneficial for slowing clinical progression.

### Replication cohort (CLIMB-DEM).

To confirm our findings, we applied the same analytic pipeline to an independent cohort of prodromal DLB patients (from the CLIMB-DEM cohort). Demographic data are summarized in Table 1. Compared with the SPIN cohort, the CLIMB-DEM cohort showed a significantly lower proportion of women (29.6% (n = 16) vs. 64.1% (n = 25) in the SPIN cohort; p-value = 0.001) and a trend toward a higher proportion of *APOEε4* carriers (43.4% vs. 23.1%, respectively; p-value = 0.071). Similar proportions of patients according to A/T scheme were observed in both cohorts. In the CLIMB-DEM cohort, CSF sTREM2 levels positively correlated with age (r = 0.289, p-value = 0.033), but no associations were found stratifying by sex (4.16 ± 1.71 in women vs. 4.15 ± 1.70 ng/mL in men, p-value = 0.987).

In line with the SPIN cohort, linear regression models, adjusted for age, showed a trend towards an association between CSF sTREM2 and Aβ42 levels (β = 0.198, p-value = 0.071), but not p-tau levels (Supplementary Tables 7). Then, we stratified patients from CLIMB-DEM cohort according to the A/T scheme. A− patients also showed a trend to higher CSF sTREM2 levels compared to A+ patients (4.54 ± 1.71 vs. 3.90 ± 1.64 ng/mL, p-value = 0.082), but no differences were observed between T− and T+ patients (4.13 ± 1.71 vs. 4.19 ± 1.67 ng/mL, p-value = 0.988, Supplementary Fig. 2). These results are aligned with those reported previously in the SPIN cohort.

Upon inclusion of APOE genotype in the linear regression model, *APOEε4* carrier status was not independently associated with CSF sTREM2 levels (β[carrier] = − 0.017, p-value = 0.893), although the direction of the effect remained consistent with the findings from the SPIN cohort (Supplementary Tables 8). Following removal of outliers (see methods section), *APOEε4* carriers exhibited significantly lower CSF sTREM2 levels compared with non-carriers (3.64 ± 1.50 vs. 4.46 ± 1.52 ng/mL, p = 0.004, Supplementary Fig. 3). In sex-stratified analyses, men *APOEε4* carriers showed a trend toward lower sTREM2 levels compared with non-carriers (3.09 ± 0.69 vs 4.35 ± 1.51 ng/mL, p-value = 0.054; n = 34), whereas no statistically significant differences were observed among women (3.42 ± 1.65 vs 4.43 ± 1.73 ng/mL, p-value = 0.204, n = 15), probable due to the low n-number of women in the CLIMB-DEM cohort (Fig. 3). Sensitivity analysis and full description of outliers are specified in the Supplementary Material (Supplementary table 9).

## Discussion

Our study advances the understanding of biological heterogeneity in DLB by investigating the interplay between TREM2-dependent microglial response, APOE genotype, and AD-related pathological changes in two clinically well-characterized cohorts with multimodal biomarker profiling and longitudinal follow-up. Progress in this field has been hindered by the scarcity of large well-characterised DLB cohorts integrating longitudinal clinical assessments with fluid and imaging biomarkers spanning early disease stages. By combining CSF biomarkers, amyloid-PET measurements, *APOE* genotyping, and longitudinal cognitive evaluations, we identified stage-dependent associations between TREM2-dependent microglial response, *APOEε4* carriage and AD-related markers across the DLB spectrum. Specifically, *APOEε4* carriage was associated with reduced CSF sTREM2 levels during the prodromal stages of DLB independently of AD-copathology, predominantly in women. Lower sTREM2 levels during the prodromal DLB stage were further related to a subsequent faster cognitive decline. We also identified a stage-dependent relationship between sTREM2 and AD core biomarkers, characterized by an early association with amyloid aggregation-related markers during prodromal DLB and a later predominance of tau-related associations in dementia stages. Our findings support a stage-specific role of TREM2-mediated microglial responses in DLB and suggest that *APOEε4* is associated with an attenuation of early microglial activation independently of AD copathology, specially in women.

First, we observed elevated sTREM2 levels in DLB groups compared to healthy volunteers, together with a differential stage-dependent relationship with AD-related markers. Interestingly, the only previous study reporting elevated CSF sTREM2 levels in DLB^37^ relied on the same in-house MSD-based immunoassay used in the present work, whereas studies using commercial immunoassays failed to detect these differences ^32,49^. Our assay additionally incorporates a neo-epitope-specific detection antibody recognizing the sTREM2 isoform specifically generated after TREM2 cleavage, and no other soluble TREM2 isoforms generated by alternative splicing ^33,46^. These results highlight the methodological sensitivity and biological specificity required for meaningful assessment of TREM2-related cell-autonomous microglial responses by the quantification of sTREM2 levels in CSF ^33^. Interestingly, prodromal DLB individuals without a CSF biomarker profile suggestive of AD copathology showed higher sTREM2 levels than amyloid-positive individuals, suggesting that early microglial response in DLB may be driven by processes beyond amyloid pathology. (Peng et al., 2020; Wilson et al., 2020). We have recently described the association between synaptic dysfunction and the subsequent longitudinal increase in CSF sTREM2 levels throughout aging independently of AD-related changes ^35^. Considering that synaptic dysfunction is an early and central event in synucleinopathies^50,51^ and accounting for our previous results, our data support that elevated sTREM2 levels in prodromal DLB may reflect an early microglial response to α-synuclein-related synaptic alterations. Consistent with this interpretation, elevated sTREM2 levels have also been reported in Parkinson’s disease ^36,52^, while atypical parkinsonian tauopathies such as progressive supranuclear palsy and corticobasal degeneration do not show similar elevations ^30^.

Interestingly, lower sTREM2 levels during prodromal DLB were associated with lower CSF Aβ42 levels and showed a trend towards an association with higher amyloid-PET uptake. These findings potentially reflect early interactions between TREM2-mediated microglial responses and amyloid aggregation, either through direct Aβ–TREM2 interactions ^31,53^ or protective microglial effects limiting early amyloid deposition ^33^. In contrast, during dementia stages, sTREM2 levels were predominantly associated with tau-related biomarkers, with higher levels observed in tau-positive individuals, showing a lack of association with amyloid-related markers. Elevated sTREM2 levels have also been reported in Tau positive PD individuals ^52^, and the cross-sectional correlation between sTREM2 and p-tau in CSF have been broadly described in different neurodegenerative disorders (Piccio et al., 2016; Suárez-Calvet et al., 2016; Suárez-Calvet et al., 2019), probably indicating a phase-dependent relationship between TREM2-dependet microglial response and neurodegeneration. Overall, these findings suggest that the relationship between sTREM2 and underlying pathological processes is stage-dependent across the DLB continuum, with early TREM2-dependent microglial response being associated with amyloid-related and probable synaptic changes and later associations increasingly driven by tau-related neurodegeneration.

One of our central findings is that *APOE* genotype influences sTREM2 levels independently of AD-related markers and specifically in prodromal DLB patients, with *APOEε4* carriers exhibiting approximately two-fold lower sTREM2 levels compared to non-carriers. This pattern contrasts with what has been observed in early stages of AD, where sTREM2 levels in *APOEε4* carriers are similar to or slightly higher than in non-carriers ^32,34^. Together with evidence from experimental models ^29,54^, our findings suggest that *APOEε4* may attenuate TREM2-mediated microglial responses to early neurodegenerative stimuli. Such an effect may be particularly evident when microglial activation is triggered by relatively subtle processes, such as synaptic dysfunction, whereas stronger stimuli, including amyloid aggregation, may override these differences at the biomarker level. This interpretation is biologically plausible given that *APOE* acts as an endogenous ligand for TREM2 ^24^ and that APOE-TREM2 signaling plays a central role in disease-associated microglial responses ^55^. Importantly, *APOE* isoforms interact differentially with TREM2 ^54,56^, with *APOEε4* inducing distinct conformational changes in the APOE–TREM2 complex (Greer et al., 2025). Consistent with our observations, *APOEε4* has been associated with delayed microglial responses to Aβ pathology in murine models, where APOE–TREM2 signalling is required for the formation of protective microglial barriers around amyloid-β plaques ^28,58^. In the context of DLB, such an early attenuation of TREM2-mediated microglial responses in *APOEε4* carriers may contribute to the greater neuropathological burden associated with this genotype, including both Lewy body pathology and Alzheimer’s copathology ^17,20,21^. Consistent with this interpretation TREM2 deficiency has been associated with aggravated α-synuclein-induced neurodegeneration and enhanced neuroinflammatory responses in animal models, supporting a protective role of TREM2-dependent response during the early stages of synucleinopathy ^59,60^.

Notably, the effect of *APOE* genotype on CSF sTREM2 levels was stronger in women. The difference in sTREM2 levels between *APOEe4* carriers and non-carriers in the SPIN cohort relied in women, while we did not find statistically significant differences in men. Further supporting this more pronounced effect on women, we found a weaker statistical association in the CLIMB-DEM cohort, with a much higher percentage of men compared to the SPIN cohort. These findings suggest a sex-dependent regulation in the APOE-TREM2 interaction. This interpretation is further supported by emerging evidence indicating that sex modulates microglial response in neurodegenerative diseases ^29,61^, with potential implications for understanding the heterogeneity observed in DLB and other related diseases. In AD, microglia from women showed greater response to amyloid-β pathology, with microglial activation mediating 57% of the amyloid-to-tau relationship in women compared to only 19% in men ^62^. Evidence from mouse models, supported by analyses of human brain tissue, indicates that *APOEε4* exerts sex-specific effects on microglial function, with females showing greater suppression of disease-associated microglial genes and a reduced transition to neuroprotective phenotypes than males. ^63,64^. Recent work has identified sex-dependent *APOEε4* neutrophil-microglia interactions that are associated with cognitive impairment in AD, with female APOEε4 carriers showing attenuated microglia responses ^61^. Moreover, sex differences have been reported across the DLB spectrum, including alterations in dopaminergic neurotransmission during prodromal stages and differences in clinical presentation (Boccalini et al., 2023; Chiu et al., 2023). Our findings suggest that differential regulation of APOE–TREM2-mediated microglial responses may represent an additional mechanism contributing to these sex-related biological and clinical differences.

Finally, our results show that higher CSF sTREM2 levels, specifically during the prodromal stage of DLB, were associated with slower cognitive decline independently of AD related markers. This stage-dependent protective effect aligns with findings in AD, where a stronger TREM2-dependent microglial response during early biological phases is associated with slower amyloid-β deposition and neurodegeneration processes, and better clinical outcomes, especially during early symptomatic phases (Ewers et al., 2019; Morenas-Rodríguez et al., 2022). The absence of this association in dementia-stage DLB may reflect either a progressive decline of TREM2-mediated microglial responses with disease progression or a transition towards a less protective microglial phenotype ^67^. This beneficial clinical effect of early microglial TREM2-dependent response found in the context of DLB is consistent with the biological framework emerging from our results. As discussed above, APOEε4 carriers exhibited an attenuation of the TREM2 response independently of AD copathology, a mechanism that may contribute to the greater pathological burden reported in this subgroup of DLB patients, further supported by the protective role of TREM2 signalling during the early stages of synucleinopathies already suggested by experimental studies ^59,60^. Together, these observations provide a biological basis for the association between higher sTREM2 levels and more favourable clinical trajectories, supporting the notion that early TREM2-mediated microglial response may represent a beneficial response capable of modulating disease progression across the DLB spectrum.

This study has several strengths. It benefits from the integration of two clinically well-characterized DLB cohorts with multimodal biomarker profiling and longitudinal cognitive follow-up, an approach that remains uncommon in Lewy body disease research. The availability of CSF biomarkers, amyloid-PET, genetic data, and longitudinal clinical assessments enabled the characterization of stage-dependent relationships between TREM2-mediated microglial responses, APOE genotype, and AD-related pathological changes, while allowing replication of the main findings across independent cohorts. Furthermore, the use of an assay specifically targeting the cleaved soluble form of TREM2 strengthens the biological specificity of the CSF measurements. Several limitations should nevertheless be acknowledged. The largely cross-sectional design precludes definitive conclusions regarding causality and the temporal dynamics of TREM2-related responses. In addition, longitudinal analyses, particularly those stratified by APOE genotype, were limited by sample size and therefore should be interpreted with caution. Finally, CSF sTREM2 represents an indirect measure of TREM2 cellular response and does not capture the full complexity of microglial states, while the sex-specific associations observed warrant confirmation in larger and more balanced cohorts.

In conclusion, our findings identify TREM2-mediated microglial response as a potential contributor to the biological and clinical heterogeneity that characterizes DLB. We show that APOEε4 carriage is associated with an attenuation of early microglial responses independently of AD copathology, particularly in women, and that lower TREM2-related response during the prodromal stage is associated to faster cognitive decline. Together, these observations support a model in which *APOE* genotype and sex interact to shape microglial responses and influence disease trajectories across the DLB spectrum. Importantly, our findings suggest that part of the effect of *APOEε4* in DLB may be mediated through neuroimmune mechanisms beyond its established relationship with AD copathology. Several therapeutic strategies aimed at enhancing TREM2 signaling are currently being evaluated in neurodegenerative disorders ^33,65^, including Alzheimer’s disease and amyotrophic lateral sclerosis. Our results suggest that the biological impact of such approaches may depend on disease stage and patient-specific characteristics, highlighting the importance of biomarker-guided stratification strategies. More broadly, our findings identify the APOE–TREM2 axis as a biologically plausible contributor to disease heterogeneity and a promising target for precision medicine approaches also in Lewy body diseases.

## Supplementary Material

This is a list of supplementary files associated with this preprint. Click to download.

• SupplementaryMaterialsZaragozaBallesterFinalsubmission.docx

## Figures and Tables

**Table 1:** Demographic and CSF biomarkers data at baseline.

	SPIN cohort				CLIMB-DEM cohort
	prodDLB (n = 39)	demDLB (n = 37)	HV (n = 40)	p- value	prodDLB (n = 53)
**Age, mean (SD), y**	74.2 (6.1) ^a †^	77.1 (4.7) ^a^	67.8 (5.2)	< 0.001	71.6 (5.6) ^†^
**Sex, women/men (% of women)**	25/14 (64.1) ^‡^	21/16 (56.8)	24/16 (60.0)	0.157	15/38 (30.1) ^‡^
**APOEε4 carriers/APOEε4 non-carriers, (% of APOEε4 carriers)**	9/30 (23.1)	12/25 (30.6)	7/33 (17.5)	0.406	23/30 (43.4)
**Age at onset, mean (SD), y**	70.8 (6.1)	73.2 (6.2)	-	0.107	68.8 (5.8)
**MMSE, mean (SD), score**	25.4 (2.7) ^b^	22.8 (4.5) ^b^	28.8 (1.2)	< 0.001	25.2 (2.2)
**Follow up, mean ± SD (range), months**	68.5 (32.9)	58.5 (30.0)	-	0.170	-
**CSF variables**					
Aβ42, mean (SD), pg/mL	753.0 (290.9)^b^	589.2 (206.6)^b^	927.5 (221.8)	< 0.001	541.8 (269.2)
A+/A−, (% of A+)	15/14 (41.7)	17/20 (45.9)	-	0.895	32/21 (60.3)
p-tau, mean (SD), pg/mL	63.8 (34.4)	65.3 (40.1)	45.3 (10.2)	0.008	61.2 (53.7)
T+/T−, (% of T+)	14/15 (34.4)	15/22 (45.9)	-	0.857	16/37 (29.6)
A + T+/non-A + T+, (% of A + T+)	8/31 (21.1)	7/30 (81.1)	-	1.000	12/41 (32.2)
t-tau, mean (SD), pg/mL	376.6 (236.3)	415.5 (316.7)	231.8 (53.4)	0.002	406.3 (205.3)
sTREM2, mean (SD), ng/mL	6.1 (2.5)	5.2 (2.9)	3.8 (1.9)	< 0.001	4.1 (1.6)
**FBP-PET**					
FBP-PET uptake, mean (SD), SUVr	1.1 (0.2)	1.2 (0.2)	-	0.177	-
Positive FBP-PET/Negative FBP-PET, (% of positive FBP-PET)	7/9 (46.7)	14/7 (66.7)	-	0.391	-

p-values for demographics are based on analysis of raw data (ANOVA/t-test for continuous variables and chi-square test for categorial variables). P-values of CSF biomarkers were based on parametric tests considering log-transformed variables, and were adjusted for sex. Positivity in the FBP-PET was defined as SUVR measurements > 1.17 p-value column represents statistical differences across SPIN cohort (prodDLB vs. demDLB vs. HV).

Superscript lowercase letters represent statistical differences between prodDLB and demDLB groups from SPIN cohort:

a p-value < 0.05

b p-value < 0.01

c p-value < 0.001.

Superscript symbols represent statistical differences between prodDLB patients from both SPIN and CLIMB-DEM cohorts:

† p-value < 0.05

‡ p-value < 0.01.

MMSE = mini-mental state examination. prodDLB = prodromal Dementia with Lewy Bodies. demDLB = mild dementia with Lewy Bodies. HV = cognitively healthy volunteers. CSF = cerebrospinal fluid. p-tau = phosphorylated tau on threonine 181. t-tau = total tau. FBP-PET=Florbetapir PET. SUVR =standardised uptake value ratio.
